# Percutaneous Transhepatic Balloon Dilation of Biliary-Enteric Anastomotic Strictures after Surgical Repair of Iatrogenic Bile Duct Injuries

**DOI:** 10.1371/journal.pone.0046478

**Published:** 2012-10-26

**Authors:** Andrew Y. Lee, John Gregorius, Robert K. Kerlan, Roy L. Gordon, Nicholas Fidelman

**Affiliations:** Department of Radiology, University of California San Francisco, San Francisco, California, United States of America; University of Colorado, United States of America

## Abstract

**Purpose:**

To evaluate the efficacy of percutaneous balloon dilation of biliary-enteric anastomotic strictures resulting from surgical repair of laparoscopic cholecystectomy-related bile duct injuries.

**Material and Methods:**

A total of 61 patients were referred to our institution from 1995 to 2010 for treatment of obstruction at the biliary-enteric anastomosis following surgical repair of laparoscopic cholecystectomy-related bile duct injuries. Of these 61 patients, 27 underwent surgical revision upon stricture diagnosis, and 34 patients were managed using balloon dilation. Of these 34 patients, 2 were lost to follow up, leaving 32 patients for analysis. The primary study objective was to determine the clinical success rate of balloon dilation of biliary-enteric anastomotic strictures. Secondary study objectives included determining anastomosis patency, rates of stricture recurrence following treatment, and morbidity.

**Results:**

Balloon dilation of biliary-enteric anastomotic strictures was clinically successful in 21 of 32 patients (66%). Anastomotic stricture recurred in one of 21 patients (5%) after an average of 13.1 years of follow-up. Patients who were unsuccessfully managed with balloon dilation required significantly more invasive procedures (6.8 v. 3.4; p = 0.02) and were left with an indwelling biliary catheter for a significantly longer period of time (8.8 v. 2.0 months; p = 0.02) than patients whose strictures could be resolved by balloon dilation. No significant differences in the number of balloon dilations performed (p = 0.17) or in the maximum balloon diameter used (p = 0.99) were demonstrated for patients with successful or unsuccessful balloon dilation outcomes.

**Conclusion:**

Percutaneous balloon dilation of anastomotic biliary strictures following surgical repair of laparoscopic cholecystectomy-related injuries may result in lasting patency of the biliary-enteric anastomosis.

## Introduction

Cholecystectomy is one of the most commonly performed elective surgical procedures in the world. In the United States alone approximately 750,000 operations are performed annually [1]. Since its introduction in the 1990s, the laparoscopic surgical approach has generally offered quicker recovery times and a lower rate of complications over the traditional open approach. However, the laparoscopic approach has also led to an estimated three to four-fold increase in the incidence of bile duct injuries [2]–[5]. Bile duct injuries occur in about 1 in every 200 patients, which amounts to approximately 4,000 cases in the United States annually [6]. Bile duct injuries are usually repaired surgically [7], [8]. The repair procedures are technically challenging, and therefore, are best performed at institutions that specialize in care for patients with bile duct injuries [9].

Surgical repair typically involves creation of a Roux-en-Y choledochojejunostomy in patients with a healthy common bile duct remnant, or a hepaticojejunostomy by connecting the common hepatic or one of the branch ducts to the jejunum when injuries are located at the liver hilum or involve one of the lobar or segmental ducts. These reparative procedures currently offer the best long-term results [10]. One of the most common complications resulting from surgical repair of laparoscopic cholecystectomy-related bile duct injuries is stricture formation at the biliary-enteric anastomosis, which follows approximately 10% of surgical repairs [11]–[13]. Traditionally, strictures at the biliary-enteric anastomosis have been approached surgically. However, surgical revision of the biliary-enteric anastomoses is complex due to build-up of extensive scar tissue at the liver hilum, difficulty with obtaining adequate exposure, and lack of adequate length of healthy bile duct needed to construct a new anastomosis.

An alternate approach to management of biliary strictures following surgical bile duct repair involves percutaneous transhepatic biliary drainage (PTBD) followed by dilation of strictures with balloons. Ideally, this minimally-invasive option offers the patient a quicker recovery and far less morbidity than surgery. There is a paucity of available data that describes clinical outcomes of patients with recurrent biliary strictures after surgical repair of iatrogenic bile duct injuries treated with balloon dilation [14]–[16]. Therefore, the purpose of this study was to describe long-term effectiveness of balloon dilation in managing strictures arising at the biliary-enteric anastomosis following surgical repair of bile duct injuries.

## Materials and Methods

### Patients

This retrospective study was approved by the Committee on Human Research of the Institutional Review Board at our institution. The requirement for informed consent for participation in the study was waived. Between March 1995 and August 2010, 61 patients (40 women and 21 men; average age at referral 52.6 years; range 23–82 years) were referred to our institution for management of biliary obstruction that developed at the biliary-enteric anastomosis created for laparoscopic cholecystectomy-related bile duct injuries. Based on the recommendation by the treating hepatobiliary surgeon, 27 of these patients underwent surgical revision, whereas 34 patients (23 women and 11 men; average age at referral 53.4 years; range 28–82 years) were treated with balloon dilation. A total of 20 patients had an injury to the common hepatic duct (7 Bismuth type 1 and 13 Bismuth type 2), while injuries at the liver hilum were sustained in 6 patients (Bismuth type 3). Three patients had injuries to the right hepatic duct (Bismuth type 4), and 3 patients had injuries to the aberrantly inserting right posterior duct draining segments 6 and 7 of the liver (Bismuth type 5). Location of injury could not be determined from the available records in 2 patients [17]. Presence or absence of a concomitant hepatic artery injury could not be ascertained based on the available records.

Strictures at the biliary-enteric anastomosis occurred following hepaticojejunostomy in 26 patients, choledochojejunostomy in 5 patients, or choledochoduodenostomy in 3 patients.. The mean time to stricture formation after surgical repair was 2.3 years (range 0–15.3 years). Mean total serum bilirubin level at time of admission was 3.9 mg/dL (range 0.8–13.5 mg/dL; normal range 0.3–1.2 mg/dL). Symptoms at admission included jaundice, chills, fever, abdominal pain, pruritus, weight loss and rigors. The average follow-up period for the study population was 13.1 years (median 11.8 years, range 1.9–17.4 years).

### Study Objectives

The primary study objective was to determine the clinical success rate of balloon dilation of biliary-enteric anastomotic strictures following surgical repair of laparoscopic cholecystectomy-related bile duct injuries. Clinical success was defined as resolution of biliary-enteric anastomotic stricture on tube cholangiogram (less than 30% residual narrowing at the biliary-enteric anastomosis when compared to the caliber of the bile duct at the cranial aspect of the anastomosis) associated with normalization of serum bilirubin (if elevated) and elimination of pruritus or other clinical symptoms (if present) allowing removal of the external biliary catheter. Secondary study objectives were to (a) evaluate patency of the anastomoses over time, (b) determine the rates of stricture recurrence following a clinically successful balloon dilation, and (c) analyze morbidity to patients associated with treatment of biliary-enteric anastomotic strictures. Duration of biliary-enteric anastomosis patency was defined as the time between removal of the external biliary catheter till documentation of stricture recurrence on a subsequent cholangiogram, a patient's death, or completion of the data analysis period (July 1, 2012). Clinical signs of stricture recurrence included jaundice, pruritus, abdominal pain, anorexia, nausea or vomiting, elevated serum bilirubin, and evidence of new or worsening intrahepatic biliary ductal dilatation on imaging (performed by contrast-enhanced CT or MRI). Due to the retrospective nature of the analysis, information on morbidity was based on the number of invasive procedures performed in the interventional radiology suite or in the operating room, the number of balloon dilation sessions or surgical operations, time period spent with an indwelling biliary catheter, number of hospitalizations for management of a bile duct-related problem (i.e. cholangitis, hemobilia), and the number and type of complications related to treatment.

The types of interventional radiology procedures tracked for the study purposes included biliary drain injections with contrast (tube check), PTBD exchanges, side-arm sheath cholangiograms, and balloon dilation sessions. If multiple interventional radiology procedures were performed on a given day, only the most complex procedure was counted. The order of procedure complexity increased from biliary drain injection with contrast (i.e. tube check; least complex) to tube exchange over a guidewire, to side-arm sheath cholangiogram (exchange of a biliary drain to a side-arm sheath over a guidewire followed by cholangiogram through the side-arm sheath; this procedure was performed to assess anastomosis patency and was preceded all balloon dilations), to balloon dilation (most complex). For example, if a patient had a drain check followed by a side-arm sheath cholangiogram, balloon dilation and PTBD replacement, the procedure was counted as “balloon dilation”. The time period spent with an indwelling biliary catheter was calculated from the day of PTBD insertion to the day of tube removal (either following successful balloon dilation or after surgical revision). Hospitalizations for bile duct-related problems that occurred after PTBD insertion were tracked as well.

### Management of Biliary-Enteric Anastomotic Strictures

**Figure 1:**
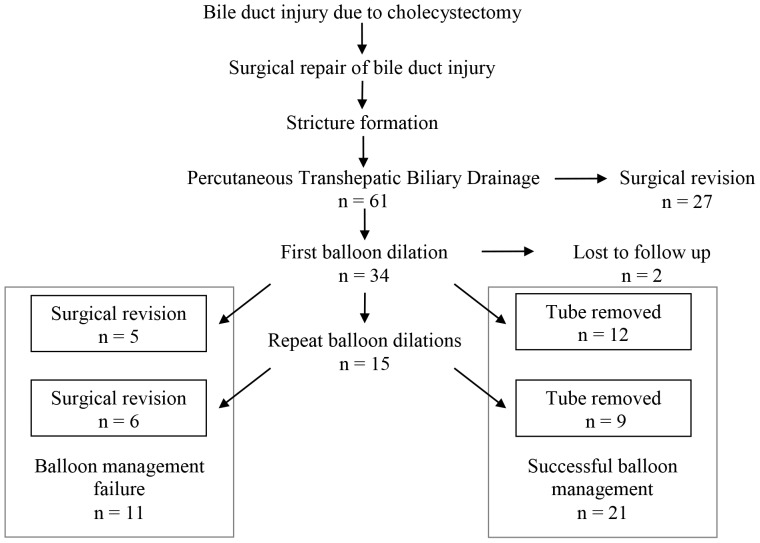
Flow chart of the study subjects (n = number of patients).

Upon referral to our institution, all patients first underwent percutaneous transhepatic cholangiography (PTC) followed by placement of a percutaneous transhepatic biliary drain (PTBD). Subsequently, 34 patients were managed with balloon dilation. Two patients were lost to follow-up (Figure 1).

**Figure 2:**
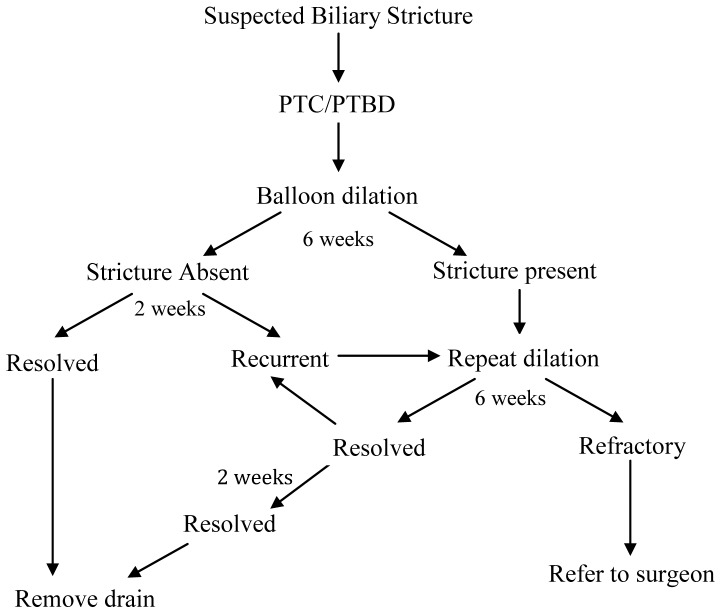
Treatment algorithm for patients with biliary anastomotic strictures who underwent at least one balloon dilation (32 patients).

Treatment algorithm for patients managed with balloon dilation is summarized in Figure 2. Balloon dilation was performed at the time of PTBD insertion or 3–14 days following drain placement. Timing of the first balloon dilation session was at the discretion of the treating interventional radiologist. Balloon dilation was performed on the same day as PTBD only if duct catheterization was technically straightforward. The first balloon dilation session was performed with 6–10 mm diameter by 40 mm angioplasty balloons (Boston Scientific, Natick MA; maximum inflation pressure 10 atm). The duration of inflation was approximately 60 seconds. Maximum balloon diameter was selected based on the caliber of the bile duct proximal to the anastomotic stricture. Anastomotic patency following balloon dilation was defined as greater than 70% of bile duct caliber proximal to the biliary-enteric anastomosis on cholangiography. Following balloon dilation, a 10.2 French internal-external biliary drainage catheter (Cook Inc., Bloomington, IN) was left in place with catheter tip in the Roux limb for a period of six weeks. At the completion of the six-week interval, patients returned for a side-arm sheath cholangiogram to assess patency of the biliary-enteric anastomosis.

Prior to 2002, if a persistent anastomotic narrowing greater than 30% of bile duct caliber was demonstrated on a follow-up cholangiogram, balloon dilation of the biliary stricture was repeated, as described above. Beginning in 2002, a repeat balloon dilation was performed with an 8–10 mm by 40 mm high-pressure balloon for 60 seconds (Conquest, Bard Inc., Murray Hill, NJ; maximum inflation pressure 24 atm) in 15 patients. After 2005, if anastomotic patency could not be restored with a high pressure balloon, a 6–8 mm by 20 mm cutting balloon (Boston Scientific) was used followed by repeat dilation with a larger diameter high-pressure balloon up to 24 atm in 2 patients. Following balloon dilation, the PTBD was replaced and was left in place for an additional six weeks after each balloon dilation session. If the anastomosis was found to be patent on the follow-up cholangiogram (less than 30% residual stenosis), a 10 French external biliary drainage catheter (Malecot, Bard) was capped and left in place above the stricture for a period of an additional two weeks. The drainage catheter was removed if the repeat tube cholangiogram demonstrated persistent patency of the anastomosis. Alternatively, recurrent or residual anastomotic stricture was treated with repeat balloon dilation (Figure 2).

At the beginning of the study period (1995–1999), patients were referred for surgical revision of the biliary-enteric anastomosis if a single attempt at balloon dilation of the anastomosis proved unsuccessful. After 2000, patients were referred to surgery after biliary strictures remained after two or more balloon dilation sessions. After the biliary drain removal, patients were instructed to return to their hepatobiliary surgeon at our institution if they experienced any recurrent biliary obstruction symptoms (jaundice, abdominal pain, fever, nausea, anorexia). Follow-up was conducted via a retrospective review of medical records and by searching the Social Security Death Index.

### Data Analysis

Statistical analysis was performed using Sas version 9.2 software (Sas Institute, Cary, IN). Continuous variables such as number of interventional radiology procedures, number of balloon dilations, maximum balloon diameter, time period with indwelling biliary catheter, and number of hospitalizations were analyzed using Student's T-test. Discrete variables, such as the number of patients who developed a recurrent stricture and individual complications were compared using Fisher's exact test. A p-value<0.05 was considered statistically significant.

## Results

Out of 61 patients referred to our institution who had previously underwent prior surgical repair for cholecystectomy-related bile duct injuries, 34 (56%) were managed with balloon dilation, and 27 patients (44%) went directly to surgical revision. Two patients treated with balloon dilation were lost to follow-up (Figure 1). During the study period 17 patients were treated with a single balloon dilation session, 11 patients underwent two balloon dilations, and 4 patients underwent three dilation sessions. A total of 15 patients with recalcitrant anastomotic strictures were treated with high-pressure balloons, and 2 patients were treated with a cutting balloon.

In 21 of the 32 patients (66%) balloon dilation proved to be clinically successful at dilating the biliary-enteric anastomotic stricture. Their clinical symptoms including jaundice, nausea, and abdominal pain resolved; serum bilirubin levels decreased from mean of 3.9 mg/dL (range 0.8–13.5 mg/dL) prior to PTBD to mean of 0.7 mg/dL (range 0.3 to 1.1 mg/dL) at the time of drain removal, while their tube cholangiograms demonstrated less than 30% residual anastomotic biliary stricture thus allowing for the biliary drainage catheter to be removed. The remaining 11 patients were initially treated with balloon dilation, but this approach was not successful in dilating the stricture after one (5 patients), two (4 patients), or three (2 patients) balloon dilation sessions. Anastomotic strictures resistant to balloon dilation were revised with Roux-en-Y hepaticojejunostomy. The decision when to proceed to surgical revision was based on the referring surgeon's preference. Prior to 2000, our institution's hepatobiliary surgeons tended to perform surgical revision of the anastomosis following a single unsuccessful dilation session, whereas after 2000 two or more balloon dilation sessions were performed prior to resorting to surgical revision.

Patients who were unsuccessfully managed with balloon dilation required significantly more invasive procedures (6.8 v. 3.4; p = 0.02) and were left with an indwelling biliary catheter for a significantly longer period of time (8.8 v. 2.0 months; p = 0.02) than patients whose strictures could be resolved by balloon dilation. No significant differences in the number of balloon dilations performed (p = 0.17) or in the maximum balloon diameter used (p = 0.99) were demonstrated for patients with successful or unsuccessful balloon dilation outcomes (Table 1).

**Table 1:** Morbidity Associated with Balloon Dilation.

	Successful (n = 21)	Unsuccessful (n = 11)	p-value
Number of IR procedures per patient [median (range)]	3 (1–9)	5 (1–15)	0.02
Sheath cholangiograms per patient [median (range)]	0 (0–1)	0 (0–1)	
Tube exchanges per patient [median (range)]	2 (0–6)	1 (0–10)	
Tube injections per patient [median (range)]	0 (0–1)	0 (0–1)	
Number of balloon dilations per patient [median (range)]	1 (1–3)	2 (1–5)	0.17
Maximum balloon diameter [mean ± standard deviation], mm	6.8±1.7	6.8±2.0	0.99
Time period with indwelling biliary catheter [mean ± standard deviation], months	2.0±1.7	8.8±11.9	0.02
Adverse events, N (%)	1 (5)	3 (27)	0.10
Additional hospitalization	1 (5)	3 (27)	
Additional hepatobiliary surgery	0 (0)	1 (9)	
Cholangitis	0 (0)	2 (18)	

After an average of 13.1 years of follow-up (range 1.9 to 17.4 years), recurrent stricture developed in one of the 21 (5%) patients who underwent a successful biliary dilation five years following the originally successful balloon dilation. This patient underwent a repeat PTBD and a single session of balloon dilation, which led to resolution of the recurrent stricture and has not required surgical revision to date (a period of 16.5 months). Thus, none of the patients who underwent a clinically successful balloon dilation ultimately developed a stricture that required surgical revision of the biliary-enteric anastomosis.

## Discussion

Laparoscopic cholecystectomy is one of the most commonly performed surgical procedures in the world, and as more of these procedures are performed, the number of patients that have sustained a bile duct injury caused by these operations has increased as well. Surgical repair of the injured duct is associated with a restricture rate of 10% to 30% [12], [18], [19]. These recurrent strictures are notoriously difficult to manage due to development of extensive scar tissue from prior surgeries at the liver hilum and have a tendency to recur at high rates. One study showed that 68% of patients who underwent surgical revision of a stricture at the biliary-enteric anastomosis following a Roux-en-Y hepaticojejunostomy developed recurrent strictures within 3 years of the most recent repair, and 80% developed a recurrence within 5 years [11]. Another study noted that about 65% of patients became symptomatic within 2 years of the last repair, and 90% of patients developed symptoms of stricture recurrence within 7 years [20]. The likely explanation for poor surgical outcome is that with each additional operation, the adhesions and scar tissue accumulate and make the surgical revisions more challenging [21].

Despite the difficulty of this clinical problem, our results demonstrate that the clinical success rate of balloon dilation was as high as 66%. All patients except one who had a clinically successful balloon dilation achieved lasting patency of the biliary-enteric anastomosis.

Previous studies have explored the possible use of percutaneous approaches to treating benign bile duct strictures, including those resulting from failed surgical repair of bile duct injuries. A study by Goykhman, et al. included 15 patients that developed biliary strictures following surgical repair for both open and laparoscopic cholecystectomy-related bile duct injuries, with 7 cases (47%) being successfully managed with balloon dilation during a mean follow up of 24 months [14]. Kocher, et al. described 21 patients who underwent balloon dilation for benign anastomotic biliary strictures that resulted from a variety of causes. Anastomotic stricture resulting from repair of iatrogenic bile duct injury was the disease etiology in 17 of the patients in that series. However, the authors did not specifically analyze patient outcomes for anastomotic biliary strictures after laparoscopic cholecystectomy [15]. A retrospective study by Misra et al. evaluated 51 patients who underwent percutaneous balloon dilation for anastomotic biliary strictures that developed following surgical repair of bile duct injuries sustained during laparoscopic cholecystectomy, and 58.8% of these patients were successfully treated (66% in our study) during a mean follow up time of 76 months. Of those patients who underwent a clinically successful balloon dilation, 76.7% did not have any recurrence of symptoms, compared to 95% in our study [16].

Our study has several limitations. First, it is a retrospective evaluation that encompasses all the problems associated with retrospective data. Our study was limited to clinical information contained in the medical records. Further evaluation with a prospective randomized trial would be needed to validate our findings. Secondly, there is the possibility that our records are not complete, and that patients developed complications after balloon dilation, but did not return to our institution. We think that likelihood of this possibility is low, as all patients in our study resided locally and could return to the care of their hepatobiliary surgeon in the event of a complication. Thirdly, as the data set encompassed patients treated over a 15-year period, the technical standards for biliary stricture dilation have changed. Namely, after the year 2000 it became standard practice to perform more than one balloon dilation session at our institution if the first balloon dilation session failed to result in biliary-enteric anastomosis patency. Subsequently, in 2002 high-pressure non-compliant balloons became available, and finally in 2005 the cutting balloons were introduced into clinical practice. Due to the small number of patients studied, it is difficult to determine whether incorporation of these new methods and technologies into standard practice had an impact on clinical outcomes. Our current institutional standard is to perform two balloon dilation sessions. During the first dilation session, a conventional balloon is used, whereas during the second dilation session, recalcitrant strictures may be subjected to dilation with a high-pressure balloon and/or a cutting balloon. If a stricture cannot be resolved following two balloon dilation sessions, the patient is referred for a surgical revision of the anastomosis.

In conclusion, our findings suggest that balloon dilation of biliary-enteric strictures resulting from bile duct injury repair may provide a lasting benefit and can prevent the need for a surgical revision of the anastomosis. We recommend that balloon dilation should be considered in patients with biliary-enteric anastomotic strictures resulting from bile duct injury repair.
